# Salvage Irradiation for Ocular Adnexal Mucosa Associated Lymphoid Tissue Lymphoma Refractory to *Chlamydia psittaci* Eradication

**DOI:** 10.1016/j.adro.2025.101822

**Published:** 2025-06-06

**Authors:** Elena Flospergher, Marianna Sassone, Anna Chiara, Fabrizio Marino, Antonio Giordano Resti, Maurilio Ponzoni, Maria Giulia Cangi, Lucia Bongiovanni, Gilda Magliacane, Giulio Modorati, Elisabetta Miserocchi, Teresa Calimeri, Piera Angelillo, Federico Erbella, Andrés J.M. Ferreri

**Affiliations:** a Lymphoma Unit, IRCCS San Raffaele Scientific Institute, Milano, Italy; b Radiotherapy and Tomotherapy Unit, IRCCS San Raffaele Scientific Institute, Milano, Italy; c Ophthalmology Unit, IRCCS San Raffaele Scientific Institute, Milano, Italy; d Pathology Unit, IRCCS San Raffaele Scientific Institute, Milano, Italy; e University Vita-Salute San Raffaele, Milano, Italy

## Abstract

**Purpose:**

Prospective trials show that *Chlamydia psittaci (Cp)* eradication with doxycycline is followed by lymphoma regression in 2-thirds of patients with ocular adnexal marginal zone lymphoma (OAMZL). Postponing orbit irradiation, a standard treatment for OAMZL, while waiting for the tumor response to antibiotic, could raise concern. Herein, we report the safety and efficacy of salvage radiation therapy in patients with OAMZL relapsed after or refractory to *Cp* eradication.

**Methods and Materials:**

Patients with stage IEA OAMZL diagnosed at our institution between 2005 and 2023 were evaluated for the safety and efficacy of radiation therapy as salvage treatment after doxycycline. Inclusion criteria were as follows: (1) first-line *Cp*-eradicating therapy with doxycycline; (2) lymphoma relapsed or progressed locally; and (3) orbital irradiation as salvage treatment.

**Results:**

A total of 28 patients (median age 66 years; range, 37-92; 16 males) were assessable; all patients but 2 (relapsing after partial response) experienced progressive disease during or after doxycycline (median 9 months; IQR, 4-40). Radiation therapy (30-36 Gy in 15-18 fractions) was well tolerated, with only 3 cases of grade-2 cataract and 3 cases of grade-1 blepharitis; all irradiated patients achieved a lymphoma regression (overall response rate = 100%), with a complete response rate of 89% (95% CI, 80%-97%). At a median follow-up of 60 months (range, 12-166) from radiation therapy, 8 patients experienced relapse, within the irradiated volume only in 2 (7%), with a 4-year progression-free survival of 74% (95% CI, 72%-75%). All patients but one are alive at a median follow-up from initial lymphoma diagnosis of 96 (IQR, 47-128) months; 22 (79%) patients are disease free.

**Conclusions:**

The postponing of orbit irradiation until relapse/progression after *Cp*-eradicating antibiotic therapy is a safe and effective strategy in patients with limited-stage OAMZL. The vast majority of patients with OAMZL can be safely managed without chemotherapeutic agents, and radiation therapy can be delayed until relapse without affecting patients’ survival.

## Introduction

Ocular adnexal marginal zone lymphoma (OAMZL) is an indolent neoplasm usually presenting as limited-stage disease, with rare cases of systemic dissemination and anecdotal tumor-related deaths.^1^ Most of these patients report a history of chronic conjunctivitis and hold *Chlamydia psittaci (Cp)* in the tumor tissue, conjunctiva, and peripheral blood mononuclear cells.^2^^,^^3^ Except for the impact of *Helicobacter pylori* in gastric mucosa-associated lymphoid tissue (MALT) lymphoma, *Cp* is the most intensively studied bacteria in MALT lymphoma and has been recognized in OAMZL as a potential target for antibiotic therapy. The prevalence of this bacteria-lymphoma association greatly ranges across reported studies; this heterogeneity has been attributed to epidemic variations across different geographical areas, differences in methods used to detect bacterial DNA or the use of wide-spectrum antibiotics before diagnostic biopsy.^4^, ^5^, ^6^, ^7^ In countries where this association has been confirmed on more series,^6^^,^^8^, ^9^, ^10^, ^11^, ^12^ bacterial eradication with specific antibiotic therapy (ie, doxycycline) has been followed by lymphoma regression in patients with both newly diagnosed and relapsed OAMZL.^9^^,^^13^^,^^14^ Whenever used as first-line treatment, 3-week doxycycline treatment has been associated with *Cp* eradication in half of patients, with an overall response rate (ORR) of 86% and a 5-year progression-free survival (PFS) of 68% for eradicated patients.^13^^,^^14^ On these grounds, 2020 ESMO Clinical Practice Guidelines reported that antibiotic therapy may be considered for initial therapy in patients with OAMZL, if they are not in need of urgent treatment to preserve their visual functionality.^15^

This antimicrobial strategy, which relies on the same principles of other antibacterial and antiviral therapies largely exploited in patients with marginal zone lymphomas, allows the avoidance of chemotherapeutic agents and the indication of radiation therapy (RT) to patients with relapsed or refractory disease. However, this later approach raises concern, because delayed RT could result in lymphoma dissemination and/or loss of chemosensitivity, mostly in patients with long-lasting stable disease after therapy with doxycycline. The adequacy, safety, and efficacy of delaying orbit irradiation until tumor progression in patients with OAMZL treated with upfront doxycycline has never been investigated.

On these grounds, we analyzed a monoinstitutional series of patients with OAMZL refractory, or only transiently responsive, to *Cp*-eradicating antibiotic therapy and irradiated to the affected orbit as salvage therapy. This study assessed response rate, duration of response, pattern of relapse, and side effects to establish whether delaying orbit irradiation until tumor progression can be applied to patients with OAMZL without impacting their survival.

## Methods and Materials

### Study population

Patients with OAMZL diagnosed at our institution between 2005 and 2023 were reviewed. Selection criteria were as follows: histologic diagnosis of OAMZL according to the most recent classifications at the time of diagnosis of each patient, stage IEA disease, first-line Cp-eradicating therapy with doxycycline, lymphoma relapsing or locally progressing after doxycycline, and orbital irradiation as salvage treatment. Histologically proven transformed forms and secondary ocular adnexal localization by marginal zone lymphomas were excluded. We identified 152 patients with OAMZL treated in the first line with doxycycline, 85 of them progressed/relapsed, 28 of them received rescue orbital RT as a second line or more advanced line (eg, after doxycycline rechallenge, intraconjunctival rituximab, high-dose clarithromycin). Given the retrospective nature of this study and anonymized clinical data, ad hoc informed consent was waived. This study conformed to the Declaration of Helsinki and was approved by the IRBs of our center.

### Baseline characteristics and therapeutic management

On diagnosis, patients underwent conventional staging work-up after encompassing: peripheral blood tests, including hepatitis virus markers and serum electrophoresis; gadolinium-enhanced magnetic resonance (MR) of the orbits; ophthalmologic examination including iconographic documentation of conjunctival lesions; contrast-enhanced computed tomography scan of the neck, thorax, and abdomen; cervical ultrasonography; breath test; bone marrow biopsy and aspirate; assessment of *Cp* infection on DNA extracted from tissue sample, conjunctival swabs and peripheral blood mononuclear cell (PBMCs) as previously reported.^13^ Patients with stage IEA-OAMZL and *Cp* infection were treated with doxycycline 100 mg every 12 hours for 3 weeks^13^ or for 4 weeks separated by 1-month interval, repeated for 3 cycles.^16^ We proposed orbit irradiation as salvage therapy to patients who experienced progressive disease during or relapsing disease after doxycycline. Irradiated patients were considered as study population. Salvage RT consisted of image guided intensity modulated RT with 6 MV photons, at a dose of 30.6-36 Gy at conventional fractionation (1.8-2 Gy/fraction; 5 fractions a week), delivered, respectively, to the affected orbit or conjunctiva. Target volume was defined on the MR imaging lymphoma lesion; the irradiation of the entire orbit or conjunctiva was avoided when possible. Response was assessed by orbit MR imaging, ophthalmologic examination, and photographs of conjunctival lesions. Response was defined according to the revised response criteria for malignant lymphoma.^17^ Radiation-related adverse events were established according to the National Cancer Institute Common Terminology Criteria for Adverse Events v5.0. The worst toxicity per organ, per patient was considered.

### Statistics

This was a retrospective, explorative study designed to establish the efficacy of RT whenever this treatment was delayed until progression/relapse after *Cp*-eradicating therapy with doxycycline. ORR to salvage RT was the primary endpoint, whereas sample size was not prospectively estimated. Duration of response, tolerability (interruptions due to toxicity), and PFS were the secondary endpoints. PFS curve was generated using the Kaplan-Meier method and was estimated as the period between initiation of RT and date of relapse, progression, death, or last follow-up visit. All analyses were carried out using the Statistica 10.0 statistical package for Windows (Statsoft Inc, 2011).

## Results

### Study population

Twenty-eight patients with stage IEA OAMZL treated with orbit RT as salvage treatment after first-line Cp-eradicating doxycycline was considered. Patient characteristics at the time of orbit irradiation are reported in Table 1. The median age of the cohort was 66 years (range, 37-92) with a male predominance (57%). Orbital soft tissues were the most common site of disease (68%) with 4 patients having bilateral lesions. Only 1 patient had MALT-IPI score over 1. At diagnosis, 18/24 (75%) patients had *Cp* infection; other infections at initial lymphoma diagnosis were gastric *H. pylori* infection in 43% of patients, eradicated in all patients but 1 before doxycycline treatment, previous hepatitis B (HBV) infection in 4 (20%) patients, and previous hepatitis C (HCV) infection in 2 (10%). Diagnosis of Sjögren syndrome was confirmed in a single patient.

**Table 1:** Patient characteristics (*n* = 28)

Findings	
Median age (range), years	66 (37-92)
Gender, *n* (%)	
Male	16 (57)
Female	12 (43)
Site of disease, *n* (%)	
Conjunctiva	8 (29)
Orbit (soft tissue)	11 (39)
Lachrymal gland	2 (7)
Orbit and conjunctiva	7 (25)
Bilateral disease	4 (14%)
MALT-IPI, *n* (%)	
0	13 (46)
1	11 (39)
2	1 (0)
Undefined	3 (11)
Other infections, *n*/*N* (%)	
*Helicobacter pylori*	9/21 (43)
Hepatitis B virus	4/20 (20)
Hepatitis C virus	2/20 (10)

### First-line and salvage treatments

All patients received first-line *Cp*-eradicating therapy with doxycycline: 9 patients received doxycycline 100 mg twice daily for 3 weeks^13^ and 19 were treated with doxycycline 100 mg twice daily for 4 weeks followed by 1 month interval, repeated for 3 cycles.^16^ All patients but 2 experienced progressive disease during (n = 11) or after (n = 15) doxycycline, with a median time to progression from doxycycline of 9 months (IQR, 4-40); the remaining 2 patients experienced lymphoma relapse after an initial partial response that lasted 14 and 58 months, respectively. At relapse/progression after doxycycline, 2 of the 10 tested patients had detectable Cp DNA in conjunctival swab and peripheral blood, respectively. All patients completed planned RT, which was well tolerated, with only 3 cases of grade-2 cataract, all treated surgically with benefit, 3 cases of grade-1 blepharitis, 1 case of grade-1 epiphora, and 1 case of grade-1 conjunctival hyperemia; all grade-1 toxicities were transient and solved without treatment interruption.

**Figure 1:**
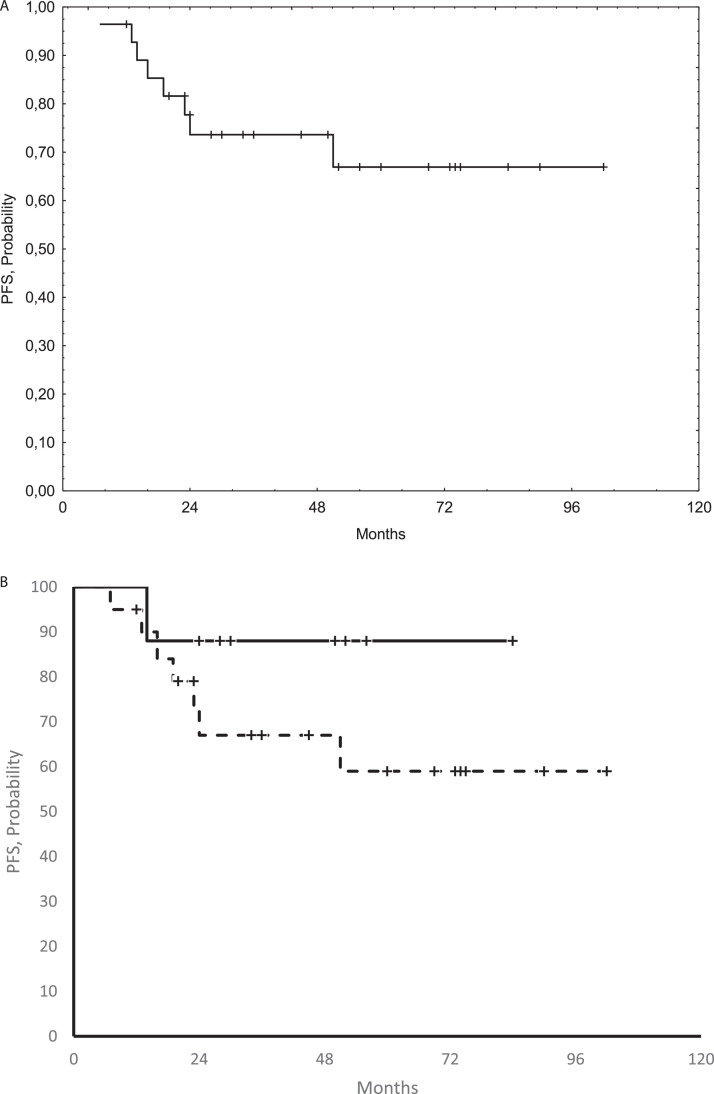
(A) Progression-free survival (PFS) of the whole study population; (B) PFS curves of the 8 patients with conjunctival lesions (continuous line) and the 20 patients with orbit ± conjunctival lesions (dotted line) treated with salvage radiation therapy (HR 4.06; 95% CI, 0.47-34.47; *P* = .19).

All irradiated patients achieved an objective response (ORR = 100%), with a complete response in 25 (89%; 95% CI, 77%-100%). At a median follow-up of 60 months (range, 12-166) after orbit irradiation, 8 patients experienced relapse: within the irradiated orbit in 2 (7%), at the contralateral orbit in 1 (4%) and at distant organs in 5 (18%), with a 5-year PFS of 67% (95% CI, 64%-70%) (Fig. 1A). Relapses after orbit irradiation were treated with 6-month doxycycline in 2 patients (2 partial responses lasting 124+ and 133+ months), clarithromycin in 3 (1 complete response lasting 152+ months and 2 partial responses lasting 11+ and 49+ months), lenalidomide plus clarithromycin in 1 (progressive disease), pulmonary irradiation in 1 (partial response lasting 49+ months), and partial resection of relapsing lesion in the palate (57+ months).

Exploratory analyses showed that age, gender, viral hepatitis, and site of disease were not associated with PFS (data not shown); however, the 8 patients with conjunctival lesions as exclusive sites of disease showed a nonsignificant trend to better outcome, with a 5-year PFS of 88% (95% CI, 87%-89%) versus 59% (95% CI, 52%-65%) for the 20 patients with orbit lesions, associated or not with conjunctival infiltration (Fig. 1B). At a median follow-up from lymphoma diagnosis of 96 months (IQR, 47-128), all patients but 1 (died due to bronchioloalveolar carcinoma) are alive, 22 (79%) are disease free, and no patient experienced ocular function alteration.

## Discussion

First-line antibiotic therapy achieves an excellent effect in patients with limited-stage, Cp-associated OAMZL. The management of failure in antibiotic therapy, as well as of early relapsing or progressive diseases, is challenging. In this study, we report for the first time the safety and efficacy of salvage ocular irradiation in a homogeneous series of patients with OAMZL refractory or only transiently responsive to *Cp*-eradicating antibiotic therapy. Through the evaluation of response rate, duration of response, pattern of relapse, and side effects, we highlight that orbit irradiation until tumor progression after doxycycline can be applied to patients with OAMZL without any negative impact on their survival. All herein considered patients achieved an objective response, with a complete response rate of 89%, similar to the 90% figure reported in series treated with upfront RT.^18^^,^^19^ After a median follow-up of 60 months since orbit irradiation, relapse occurred in a few patients, more often at distant, nonirradiated organs or at the contralateral orbit (21% of cases), reflecting patterns and rates similar to those previously published in OAMZL series treated with first-line RT (25%).^14^ In line with previously reports,^18^, ^19^, ^20^ relapses occurred more commonly among patients with orbit and/or lacrimal gland lesions. In the present study, delaying RT until relapse after Cp-eradicating antibiotic therapy did not require the use of higher radiation doses to improve local disease control. Thus, post-actinic toxicity in the present series compares favorably with published data, with only mild acute side effects without any records of long-term toxicity or sequela.

This study shows some limitations that deserve to be addressed. Given its retrospective nature, selection biases may occur. In particular, it is difficult to report the results with other salvage therapies (ie, clarithromycin, lenalidomide, antiviral therapies) used in other patients with OAMZL unresponsive to upfront doxycycline. However, we consider that patients with the more severe characteristics (eg, large lesion causing exophthalmos or other relevant symptoms) at relapse were referred to RT, which should avoid *bona fide* any risk of overestimation of efficacy of ocular irradiation.

The overall prognosis of patients with limited-stage OAMZL is among the best ones achieved in lymphomas, with rare cases of systemic dissemination and anecdotal tumor-related deaths. In this favorable scenario, the excellent activity of RT^18^, ^19^, ^20^, ^21^, ^22^ should be put in context, considering also its related acute and chronic toxicities. Long-term complications of orbit irradiation are dose-dependent and predominantly consist of increased risk of cataract and dry eyes for doses >10 Gy, whereas ischemic retinopathy, optic atrophy, corneal ulceration, and glaucoma are uncommon for doses below 36 Gy. The reported incidence of radiation-related late sequelae was 29%-45% in patients treated with doses of 24-30 Gy.^18^^,^^23^^,^^24^ Despite offering excellent local control, RT may be inadequate in preventing systemic relapse, because it occurs in 13% to 25% of patients (median follow-up between 58 and 73 months).^18^, ^19^, ^20^^,^^23^^,^^24^ These issues suggested us to postpone orbit irradiation after failure of Cp-eradicating antibiotics; according to our experience, this approach was feasible, safe, and effective, without negative impact on survival and organ functionality.

In the present study, a homogeneous radiation dose was administered and therefore it is not possible to provide a recommendation about the most effective dose. Some studies reported that ultra-low-dose RT with 4 Gy (2 Gy × 2) is effective and well tolerated in patients affected with indolent non-Hodgkin lymphoma, including MALT lymphoma,^23^^,^^24^ with a better toxicity profile and equivalent local control rate in comparison with standard doses in OAMZL.^25^^,^^26^ A recent single-arm phase 2 trial addressed a strategy adapted to the response achieved after ultra-low-dose RT: all patients received 4 Gy in 2 fractions, no additional irradiation was indicated in patients who achieved a complete remission after 4 Gy, whereas patients with persistent orbital lymphoma were offered an additional 20 Gy in 10 fractions.^26^ With limited follow-up, this strategy conferred high local control rates and spared most patients from full doses of RT.^26^ Noticeably, all these experiences, like other recent studies supporting “watch and wait” strategy^27^ or the addition of rituximab to orbit irradiation,^20^ regard upfront ocular irradiation used in retrospective, often monoinstitutional series. Thus, future studies will be needed to determine whether these concepts can be applied to patients with relapsed or progressed OAMZL.

## Conclusion

This study further contributes to the concept that patients with OAMZL can be managed with antimicrobial therapies, indicating conventional chemotherapy and RT to selected patients with locally invasive, disseminated, or relapsed disease. In particular, postponing orbit irradiation until relapse/progression after *Cp*-eradicating antibiotic therapy is a well-tolerated and effective strategy in patients with limited-stage OAMZL.

## Declaration of AI and AI-Assisted Technologies in the Writing Process

No AI technologies have been used to write this article.

## Disclosures

None.

## References

[1] Sassone M., Ponzoni M., Ferreri AJ. (2017). Ocular adnexal marginal zone lymphoma: Clinical presentation, pathogenesis, diagnosis, prognosis, and treatment. Best Pract Res Clin Haematol.

[2] Dolcetti R., Serraino D., Dognini G. (2012). Exposure to animals and increased risk of marginal zone B-cell lymphomas of the ocular adnexae. Br J Cancer.

[3] Ferreri A.J., Dolcetti R., Dognini G.P. (2008). *Chlamydophila psittaci* is viable and infectious in the conjunctiva and peripheral blood of patients with ocular adnexal lymphoma: Results of a single-center prospective case-control study. Int J Cancer.

[4] Decaudin D., Dolcetti R., de Cremoux P. (2008). Variable association between Chlamydophila psittaci infection and ocular adnexal lymphomas: Methodological biases or true geographical variations?. Anticancer Drugs.

[5] Ferreri A.J., Dolcetti R., Magnino S., Doglioni C., Ponzoni M. (2009). Chlamydial infection: The link with ocular adnexal lymphomas. Nat Rev Clin Oncol.

[6] Carugi A., Onnis A., Antonicelli G. (2010). Geographic variation and environmental conditions as cofactors in Chlamydia psittaci association with ocular adnexal lymphomas: A comparison between Italian and African samples. Hematol Oncol.

[7] Chanudet E., Zhou Y., Bacon C.M. (2006). Chlamydia psittaci is variably associated with ocular adnexal MALT lymphoma in different geographical regions. J Pathol.

[8] Ferreri A.J., Guidoboni M., Ponzoni M. (2003). Evidence for association between Chlamydia psittaci infection and ocular adnexal lymphoma (OAL). Proc Annu Meet Am Soc Clin Oncol.

[9] Yoo C., Ryu M., Huh J. (2007). Chlamydia psittaci infection and clinicopathologic analysis of ocular adnexal lymphomas in Korea. Am J Hematol.

[10] Portlock C.S., Hamlin P., Noy A. (2008). Infectious disease associations in advanced stage, indolent lymphoma (follicular and nonfollicular): Developing a lymphoma prevention strategy. Ann Oncol.

[11] Ponzoni M., Ferreri A.J., Guidoboni M. (2008). Chlamydia infection and lymphomas: Association beyond ocular adnexal lymphomas highlighted by multiple detection methods. Clin Cancer Res.

[12] Aigelsreiter A., Leitner E., Deutsch A.J. (2008). Chlamydia psittaci in MALT lymphomas of ocular adnexals: The Austrian experience. Leuk Res.

[13] Ferreri A.J., Govi S., Pasini E. (2012). Chlamydophila psittaci eradication with doxycycline as first-line targeted therapy for ocular adnexae lymphoma: Final results of an international phase II trial. J Clin Oncol.

[14] Ferreri A.J., Ponzoni M., Guidoboni M. (2006). Bacteria-eradicating therapy with doxycycline in ocular adnexal MALT lymphoma: A multicenter prospective trial. J Natl Cancer Inst.

[15] Zucca E., Arcaini L., Buske C. (2020). Marginal zone lymphomas: ESMO Clinical Practice Guidelines for diagnosis, treatment and follow-up. Ann Oncol.

[16] Ferreri A.J., Sassone M.C., Cangi M.G. (2022). Six-month doxycycline is safe and effective as upfront monotherapy for stage-I MALT lymphoma of the ocular adnexae: Primary endpoint results of the IELSG39 trial. Blood.

[17] Cheson B.D., Pfistner B., Juweid M.E. (2007). Revised response criteria for malignant lymphoma. J Clin Oncol.

[18] Goda J.S., Le L.W., Lapperriere N.J. (2011). Localized orbital mucosa-associated lymphoma tissue lymphoma managed with primary radiation therapy: Efficacy and toxicity. Int J Radiat Oncol Biol Phys.

[19] Bayraktar S., Bayraktar U.D., Stefanovic A., Lossos IS. (2011). Primary ocular adnexal mucosa-associated lymphoid tissue lymphoma (MALT): Single institution experience in a large cohort of patients. Br J Haematol.

[20] Hashimoto N., Sasaki R., Nishimura H. (2012). Long-term outcome and patterns of failure in primary ocular adnexal mucosa-associated lymphoid tissue lymphoma treated with radiotherapy. Int J Radiat Oncol Biol Phys.

[21] Niwa M., Ishikura S., Tatekawa K. (2020). Radiotherapy alone for stage IE ocular adnexal mucosa-associated lymphoid tissue lymphomas: Long-term results. Radiat Oncol.

[22] Uno T., Isobe K., Shikama N. (2003). Radiotherapy for extranodal, marginal zone, B-cell lymphoma of mucosa-associated lymphoid tissue originating in the ocular adnexa: A multiinstitutional, retrospective review of 50 patients. Cancer.

[23] Pereira-Da Silva M.V., Di Nicola M.L., Altomare F. (2022). Radiation therapy for primary orbital and ocular adnexal lymphoma. Clin Transl Radiat Oncol.

[24] Hoskin P., Popova B., Schofield O. (2021). 4 Gy versus 24 Gy radiotherapy for follicular and marginal zone lymphoma (Fort): Long-term follow-up of a multicentre, randomised, phase 3, non-inferiority trial. Lancet Oncol.

[25] Fasola C.E., Jones J.C., Huang D.D. (2013). Low-dose radiation therapy (2 Gy x 2) in the treatment of orbital lymphoma. Int J Radiat Oncol Biol Phys.

[26] Pinnix C.C., Dabaja B.S., Gunther J.R. (2024). Response-adapted ultralow-dose radiation therapy for orbital indolent B-cell lymphoma: A phase 2 nonrandomized controlled trial. JAMA Oncol.

[27] Mizuhara K., Kobayashi T., Nakao M. (2023). Watchful waiting is an acceptable treatment option for asymptomatic primary ocular adnexal mucosa-associated lymphoid tissue lymphoma: A retrospective study. Cancer Med.

